# Genomic analysis reveals broad adaptability of coral-killing sponge (*Terpios hoshinota*) under environmental stress

**DOI:** 10.1186/s12864-025-11962-7

**Published:** 2025-09-26

**Authors:** Po-Yu Liu, Wei-Chih Chiu, Sim Lin Lim, Hsing-Ju Chen, Yu-Hsiang Chen, Hsiang-Iu Wang, Cheng-Yu Yang, Chi-Chun Chen, Yoko Nozawa, Hideyuki Yamashiro, Kazuhiko Sakai, Sen-Lin Tang

**Affiliations:** 1 School of Medicine, College of Medicine, National Sun Yat-sen University, Kaohsiung, 804201 Taiwan; 2 Department of Biomedical Science and Environmental Biology, Kaohsiung Medical University, Kaohsiung, 807378 Taiwan; 3 Biodiversity Research Center, Academia Sinica, Taipei, 115201 Taiwan; 4 Department of Bioscience and Biotechnology, National Taiwan Ocean University, Keelung, 202301 Taiwan; 5 International Institute for Sustainability with Knotted Chiral Meta Matter (WPI-SKCM²), Hiroshima University, 1-3-1 Kagamiyama, Higashi-Hiroshima, Hiroshima, 739-8526 Japan; 6 Tropical Biosphere Research Center, Sesoko Station, University of the Ryukyus, Sesoko, Motobu-cho Okinawa, 3422, 905-0227 Japan

**Keywords:** *Terpios Hoshinota*, Coral-killing sponge, Genome assembly, Phylogenomics, Invasive species, Climate stress adaptation, Coral reef ecology

## Abstract

The coral-killing sponge, *Terpios hoshinota*, poses a significant ecological threat to coral reefs, exhibiting rapid expansion and competitive overgrowth. Despite its invasiveness, the genomic basis underlying its adaptability and resilience remains largely unexplored. Here, we present a high-quality genome assembly of *T. hoshinota*, comprising 169.4 Mb with 40,945 predicted genes. Phylogenomic analysis estimated its divergence from other demosponges during the Ordovician (~ 471 million years ago), even though its simple morphology suggests a more ancient evolutionary origin. Comparative genomic analyses revealed enrichment of genes related to substrate adhesion, innate immunity, and developmental pathways, including expansions of Wnt signaling, homeobox genes, and cell migration gene ontologies which may contribute to its aggressive growth and resilience. Transcriptomic responses under simulated climate stress conditions (heat stress at 31 °C and acidification at 700 ppm pCO₂) indicated dynamic gene regulation, with upregulation of neurotransmitter metabolism, cellular maintenance, and ion homeostasis responses. Despite these stressors, it remained stable. This suggests that *T. hoshinota* exhibits strong adaptability and resilience through rapid gene regulation. In conclusion, these findings provide molecular insights into *T. hoshinota*’s ecological success, its potential expansion under climate change, and its broader impact on coral reef ecosystems.

**Supplementary Information:**

The online version contains supplementary material available at 10.1186/s12864-025-11962-7.

## Introduction

*Terpios hoshinota* (Rützler & Muzik, 1993) (order Hadromerida and family Suberitidae), commonly known as the black encrusting sponge, is a dark gray, encrusting sponge with a radial arrangement of oscula. Its ostia (inhalant pores) are inconspicuous, and its body thickness is only approximately 1–2 mm, while its surface area varies, with some individuals reaching several meters in diameter. This species was first reported in Guam by Bryan et al. (1973). Notably, *T. hoshinota* competes with corals for space by overgrowing them, ultimately leading to coral mortality [1]. When sponge populations become excessive, the number of corals affected increases substantially [2]. During the 1980 s, an outbreak of *T. hoshinota* in Okinawa, Japan, resulted in significant coral mortality. Local newspapers referred to this phenomenon as “black disease” [3]. In 2005–2006, large populations of *T. hoshinota* were also recorded at Green and Orchid Islands, Taiwan, where they similarly caused widespread coral death [4]. More than 30% of corals died in certain reef areas at both islands. Over the past 15 years, recurrent outbreaks of *T. hoshinota* have been reported at coral reefs in the western Pacific and Indian Oceans, leading to extensive coral mortality. This sponge has been identified as a major driver of coral decline in some islands [5–16]. Notably, recent outbreaks have also been observed around Taiping Island (Spratly Islands), Taiwan [15], where *T. hoshinota* coverage in certain reef areas has reached as much as 60%, leading to significant coral loss. Furthermore, coral recovery following these outbreaks is often slow, requiring extended periods, and in some areas, no signs of recovery have been observed even after more than a decade. As a result, overgrowth of *T. hoshinota* is now recognized as a significant threat to coral survival.

*T. hoshinota* is a typical cyanobacteriosponge characterized by grayish-black encrustations containing abundant, unicellular cyanobacteria [3]. These symbionts produce three key photosynthetic pigments—chlorophyll *a*, phycocyanin, and R-phycoerythrin—enabling efficient absorption across the full visible light spectrum [17]. Regardless of the host coral species, cyanobacteria consistently dominate the microbial community of *T. hoshinota*, comprising 61–98% of the bacterial population. Notably, the cyanobacteria associated with *T. hoshinota* are phylogenetically distinct from those associated with corals, suggesting a high degree of host specificity [18]. A global survey of *T. hoshinota*-associated microbiota further identified *Proteobacteria* and *Cyanobacteria* as the predominant symbionts, together accounting for 84.1–97.9% of the community. Among them, a dominant cyanobacterial symbiont, *Candidatus* Paraprochloron terpiosi LD05, has been sequenced, revealing a metabolic potential indicative of active roles in carbon, nitrogen cycling, and vitamin B biosynthesis, thus underscoring its functional importance in environmental adaptation and even in sponge–coral competition [19].

Relationships between *T. hoshinota*-cyanobacteria and corals remain unclear. Its capacity for coral killing appearsrelated to habitat space competition. This hypothesis is supported by field observations [2] and an isotope analysis with physiological assessment, indicating that coral mortality is primarily driven by competition for space and light, mediated by shading and release of allelopathic compounds, rather than by direct predation [20]. This aggressiveness is probably facilitated by photosynthesis of symbiotic cyanobacteria and nitrogen assimilation or toxin production [19, 21]. Nevertheless, not all corals are defenseless. In at least some circumstances, *Montipora aequituberculata* resists or even outcompetes overgrowth by *T. hoshinota* [22, 23].

Regarding changes in prevalence of sponges in coral reefs, climate change-driven increases in seawater temperature and acidification have been widely recognized as major stressors impacting coral reef ecosystems. Sponges, as integral components of these ecosystems, are likewise affected by these environmental changes [24]. Under acidic conditions, calcification of corals is significantly impaired, leading to a reduction in skeletal integrity and growth rates, particularly in species that rely on calcium carbonate-based structures [25, 26]. Elevated seawater temperatures not only disrupt the physiology of marine organisms, but also destabilize their symbiotic microbial communities, thereby compromising their immune defenses and increasing their susceptibility to pathogens [27–29]. Furthermore, ocean warming and acidification create conditions that may favor proliferation of opportunistic marine pathogens, heightening the risk of disease outbreaks in coral reef ecosystems [24]. For example, *Terpios hoshinota* has shown increased aggression against *Montipora digitata* during summer at Sesoko Island, suggesting that future ocean conditions may enhance its competitive advantage versus corals [30].

Even though ecological studies on *Terpios hoshinota*, especially its invasive ecology, have provided critical insights for coral reef conservation [7, 12, 31, 32], its genomic architecture remains largely unexplored. In late 2024, a highly fragmented draft genome was released (GenBank ID: GCA_041731275.1); however, the absence of an accompanying journal article or comprehensive functional annotations has significantly limited our understanding of its unique genetic traits and morphological features. In order to understand the structural basis for the invasiveness of *T. hoshinota*, it is necessary to develop molecular insights into genes related to skeletal formation and tissue organization. Our analysis targets silicatein, a key enzyme involved in spicule formation, which is crucial for structural integrity of demosponges and underscores its role in skeletal development [33–36]. Likewise, developmental signaling pathways are also important. For instance, the ancestral Wnt signaling pathway, a fundamental regulator of cell polarity and axis formation, mediates developmental processes among basal metazoans [37]. Moreover, homeobox gene duplications are associated with increased morphological complexity in sponges and other metazoans, emphasizing their role in body plan evolution [38, 39]. Beyond development, immune-related genes such as those encoding Toll/interleukin-1 receptor (TIR), NOD-like receptors (NLR), and scavenger receptor cysteine-rich (SRCR) domains are critical for pathogen recognition and symbiotic interactions, forming an essential part of immune defense in filter-feeding invertebrates [40–42]. Taken together, these molecular features provide a foundation for exploring genetic adaptations that contribute to the ecological success and resilience of *T. hoshinota*. To this end, we also examined its transcriptional responses to climate-induced stressors, highlighting potential risks of future population expansions and their broader ecological impact on coral reef ecosystems.

## Materials and methods

### Sample collection and experiment design

**Fig. 1:**
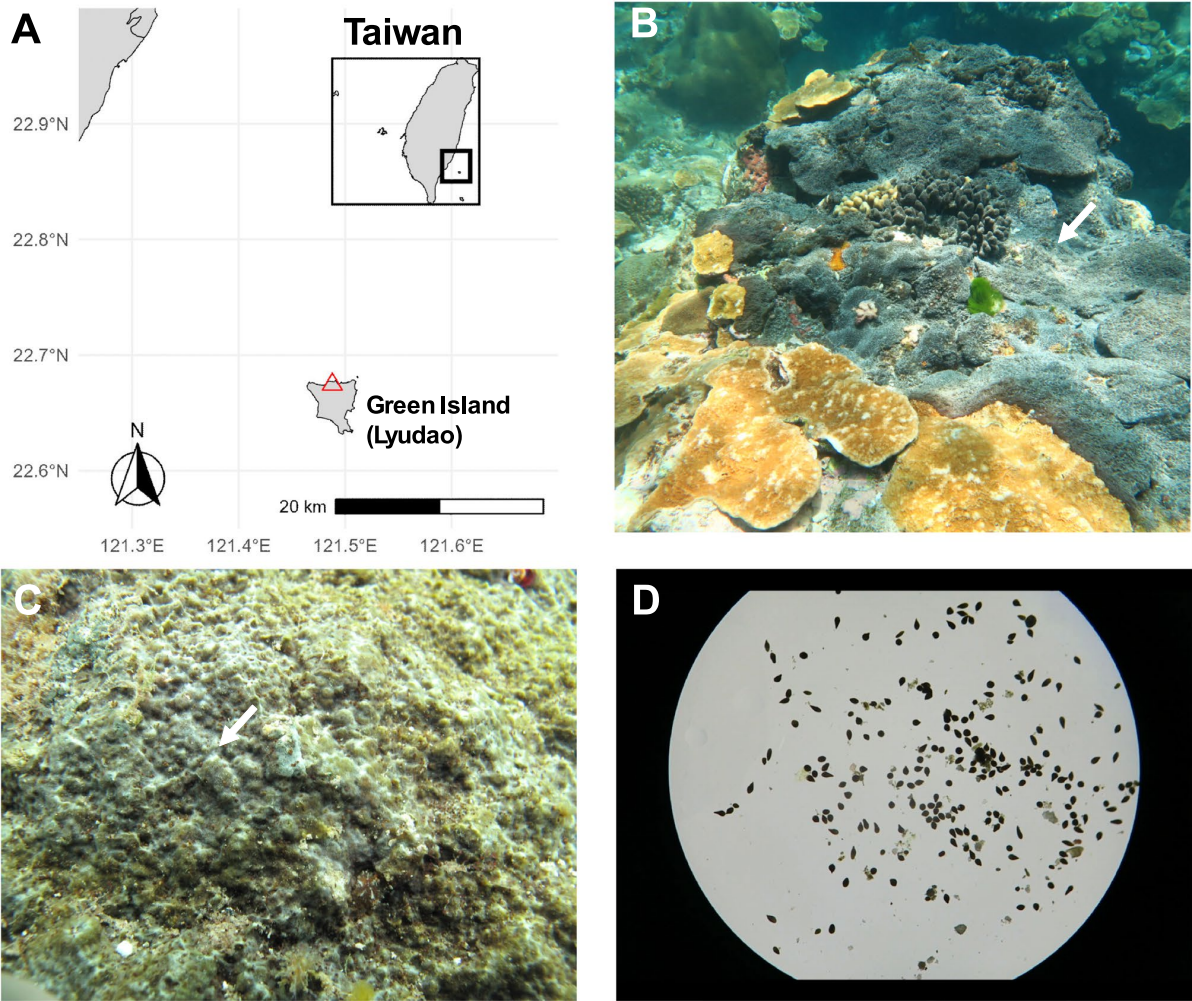
Sampling location and morphology of *Terpios hoshinota* **(A)** Sampling site at Gongguan, Green Island (Lyudao), Taiwan (22°40.29’N; 121°29.17’E), where *T. hoshinota* larvae were collected over five consecutive days in July 2020 through SCUBA diving. **(B)** *T. hoshinota* (gray) covering corals, demonstrating its invasive interaction and threat to reef ecosystems. **(C)** A close-up of a brown spot on the sponge colony was examined, and larvae were collected using a dropper and transferred into a 50 mL Falcon tube. **(D)** Genome sequencing utilized *T. hoshinota* larvae to minimize contamination from other organisms

Sponge larvae were collected over 5 consecutive days in July 2020 through SCUBA diving at Gongguan, Green Island (Lyudao), Taiwan (22°40.29’N; 121°29.17’E) (Fig. 1). Spawning behavior of sponge larvae was observed, and individual *T. hoshinota* releasing substantial numbers of larvae were identified. Larvae were collected using a pipette.

Immediately after collection, larvae were transported to the laboratory for processing. Under a stereo microscope, they were carefully inspected and isolated to prevent contamination with debris or other organisms. A total of 900 larvae were collected, preserved in absolute ethanol, and stored at −20 °C until DNA extraction.

### Genome size determination using flow cytometry

*T. hoshinota* tissue samples were collected from Chaikou, Green Island, on July 2014. Samples were fixed in absolute ethanol and stored at 4 °C until use. Prior to analysis, excess ethanol was removed by gently blotting samples with Kimwipes. Fresh *Oryza sativa* (rice) leaves were collected on the day of the analysis as a control.

Small fragments of sponge tissue and rice leaves were placed in petri dishes containing 400 mL of PI working solution. Samples were finely chopped using a scalpel. Sponge and rice suspensions were resuspended in 2 mL of working PI solution, which consisted of Tris-MgCl₂ buffer (200 mM Tris, 4 mM MgCl₂·6 H₂O, 0.5% (v/v) Triton X-100, pH 7.5) with 0.4% PVP-40. PI solution and RNase A were added to achieve a final concentration of 50 µg/mL for both. The suspension was filtered through a 20-µm filter prior to flow cytometry.

Nucleic acid content was analyzed using a 488-nm laser under conditions in which cell counts > 100,000 cells. Experiments were performed in triplicate, and mean values were calculated. The analysis specifically targeted sponge nuclei, but could not entirely exclude interference from autofluorescent symbionts. The primary autofluorescent symbionts in *T. hoshinota* were cyanobacteria, as confirmed by PCR, which showed minimal contamination in the sorted solutions. In addition, to evaluate cyanobacterial contamination in the sorted nucleus-like particle (NLP) solution, PCR amplification was performed using a cyanobacterium-specific 16S rRNA primer pair: CYA106F (5’-CGGACGGGTGAGTAACGCGTGA-3’) and CYA781R (5’-GACTACWGGGGTATCTAATCCCWTT-3’), targeting a 675-bp fragment. PCR cycling conditions included an initial denaturation at 94 °C for 3 min, followed by 30 cycles of 94 °C for 30 s, 55 °C for 30 s, and 72 °C for 1 min, with a final extension at 72 °C for 5 min. Gel electrophoresis results showed that cyanobacterial DNA was either undetectable or present at minimal levels in the NLP solution (see Supplementary Figure S2), confirming low levels of cyanobacterial contamination.

### Nanopore genome sequencing

T. *hoshinota* larvae collected from Gongguan, Green Island were transferred to a microcentrifuge tube and resuspended in 1 ml 1X TE buffer (10 mM Tris-HCl; Sigma-Aldrich, USA, pH 8.0; 1 mM EDTA; Sigma-Aldrich, USA). After 5 min centrifugation (13,000 rpm), the supernatant was discarded. Total DNA was extracted using a modified CTAB method [18]. The pellet was resuspended in 570 µL TE buffer, 30 µl 10%(w/v) SDS (Sigma-Aldrich, USA), and 5 µL 100 mg/ml RNase A (QIAGEN, USA), followed by vortexing and incubating at 37 °C for 1 h to lyse cells and degrade RNA. 3 µL 20 mg/ml protease K (New England Biolabs, USA) was then added and incubated at 50 °C for 30 min, followed by the addition of 100 µL of 5 M NaCl (Sigma-Aldrich, USA) and 80 µL of CTAB/NaCl solution, i.e., 4% NaCl; Sigma-Aldrich, USA and 10% CTAB; Sigma-Aldrich, USA. Samples were incubated at 65 °C for 10 min, and the supernatant was then transferred into sterilized tubes and treated with 600 µL of chloroform/isoamyl alcohol (24:1; Sigma-Aldrich, USA) solution. Samples were centrifuged at 13,000 rpm for 5 min at 4 °C. The upper aqueous phase was transferred to a fresh tube with addition of an equal volume of phenol/chloroform/isoamyl alcohol (25:24:1; Sigma-Aldrich, USA). Samples were centrifuged at 13,000 rpm for 5 min at 4 °C, and supernatant was transferred to a new tube. The previous step was then repeated. DNA was precipitated with 300 µL isopropanol (Sigma-Aldrich, USA) followed by centrifuging at 13,000 rpm for 10 min, after which the supernatant was discarded. The pellet was washed with 300 µL 70% pre-chilled ethanol (Sigma-Aldrich, USA), air-dried, and resuspended in 20µL sterilized Milli-Q water.

Genomic DNA samples were assessed for concentration and purity using a Qubit 2 Fluorometer (Thermo Fisher Scientific, USA) in combination with a Quant-iT™ dsDNA HS Assay Kit (Thermo Fisher Scientific, USA), Samples meeting library preparation standards were processed with an SQK-LSK109 Ligation Sequencing Kit and sequenced on an Oxford Nanopore GridION. High-quality DNA from *T. hoshinota* larvae was sequenced using two flow cells, yielding 7.58 million reads with an average N50 read length of 5.52 Kb and a total data output of 25.25 Gb (Table 1). Sequencing was conducted at the NGS High Throughput Genomics Core, Biodiversity Research Center, Academia Sinica.

**Table 1:** Sequencing statistics from the *T. hoshinota* genome

Sample source	Sample ID	Avg. Lib. Length (bp)	Dataset ID	Run no.	Reads (M)	Estimated N50 (Kb)	Passed Bases (Gb)	Total Bases (Gb)
*T. hoshinota* larvae	SG20-BQ01 SREXS-1	11,620	nd014	1	3.19	5.61	10.87	25.25
				2	0.97	5.93	3.34	
	SG20-BQ01 SREXS-2	10,301	nd017	3	2.86	5.14	9.26	
				4	0.56	5.41	1.78	

### Genome assembly, annotation

ONT sequencing reads were assembled using the Flye assembler [43, 44], employing high-quality read mode (--nano-hq). To enhance assembly accuracy, initial assemblies were iteratively polished four times with Pilon [45] by aligning raw reads using BWA-MEM2 [46]. Assembly quality was assessed with Quast [47]. Since sequencing included DNA from symbiotic cyanobacteria, the eukaryotic binning tool EukRep (-k 7 --model linsvm_160_7mer_1.0.pickle --tie euk) [48] was used to differentiate eukaryotic and prokaryotic contigs. Following initial binning, we conducted manual curation by functional annotations of contigs, and removed contigs that contained bacterial genes. Only one contig was removed. The quality of the eukaryotic contigs was then evaluated using BUSCO [49, 50] to determine completeness and contamination metrics.

**Fig. 2:**
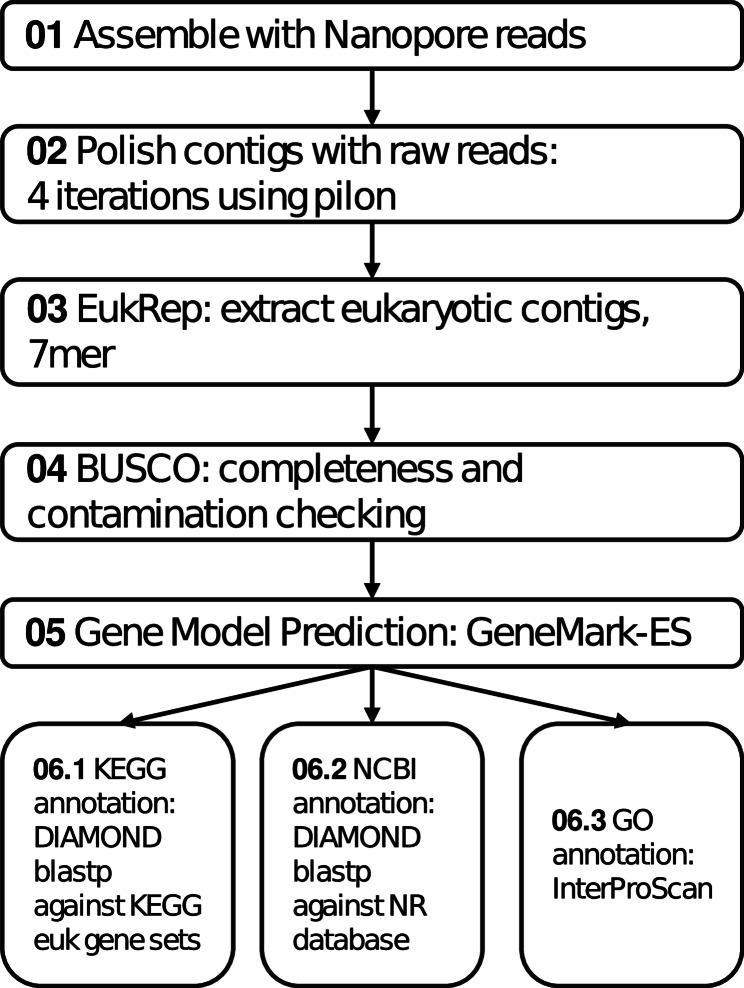
Workflow for *T. hoshinota* genome assembly and annotation The genome was assembled using Nanopore reads and polished by four iterations with Pilon. Eukaryotic contigs were then extracted using EukRep, and genome completeness was evaluated with BUSCO. Gene models were predicted using GeneMark-ES and annotated using the KEGG, NCBI NR, and GO databases

Gene prediction was initially performed using GeneMark-ES, followed by BRAKER-3 (v3.0.8) with RNA-seq data to improve gene model accuracy. Predicted protein sequences were used for downstream functional annotation. DIAMOND blastp (-e 1e^−5^ --max-target-seqs 1) searches were conducted against the KEGG database (eukaryote dataset, retrieved in 2022.07) to assign KEGG Orthology, protein names, and closest species matches. Annotation was further refined using the NCBI NR database (retrieved in 2022.12) by DIAMOND blastp (-e 1e^−5^ --max-target-seqs 1). Functional domains of proteins were predicted using InterProScan (-f tsv --goterms -dp) [51], and gene ontology (GO) terms were assigned for functional classification. Assembly and annotation workflow are described in Fig. 2.

### Phylogenomic analysis

Twelve sponge protein sequences, including *Amphimedon queenslandica* (GCA_000090795.2), *Geodia barretti* (GCA_030341885.1), *Leucosolenia complicata* [52], *Oscarella pearsei* [53], *Stylissa carteri* [54], *Sycon ciliatum* (GCA_964019385.1), *Tethya wilhelma* (GCA_964030475.1), *Xestospongia testudinaria* [54], *Corticium candelabrum* (GCA_963422355.1), *Dysidea avara* (GCA_963678975.2), *Halichondria panicea* (GCA_963675165.1), and *Oscarella lobularis* (GCA_947507565.1), were included for phylogenomic analysis with OrthoFinder (-t 64 -a 16 -M msa) using protein sequences [55–58] and inferring a species tree employing the STAG method with 108 orthogroups.

To investigate the evolutionary timescale of *Terpios hoshinota* in a broader phylogenomic framework, we used OrthoFinder [55–58] to identify single-copy orthologous genes. We analyzed the genome of *T. hoshinota* and reference genomes of *Acropora digitifera* (GCA_000222465.2), *Amphimedon queenslandica* (GCA_000090795.2), *Danio rerio* (GCA_000002035.4), *Drosophila melanogaster* (GCA_000001215.4), *Homo sapiens* (GCA_000001405.29), *Hydra vulgaris* (GCA_038396675.1), *Mus musculus* (GCA_000001635.9), *Nematostella vectensis* (GCA_932526225.1), *Tribolium castaneum* (GCA_031307605.1), *Trichoplax adhaerens* (GCA_000150275.1), *Monosiga brevicollis* (GCA_000002865.1), and *Salpingoeca rosetta* (GCA_000188695.1). A total of 20 shared single-copy genes were identified and then concatenated to perform phylogenetic analysis using the Maximum Likelihood (ML) method, with the JTT matrix-based substitution model. A Timetree analysis was performed using the RelTime method in MEGA 11 [59]. Divergence times were estimated with three calibration constraints: *Homo sapiens*-*Mus musculus* divergence (87 MYA, CI: 81.3–91.0 MYA), *Homo sapiens* - *Danio rerio* divergence (429 MYA, CI: 423.3–440.0 MYA), and *Hydra vulgaris–Acropora digitifera* divergence (571 MYA, CI: 543.0–610.0 MYA) which were retrieved from TimeTree5 (https://timetree.org/) [60]. Confidence intervals for divergence times were computed using the Tao et al. method [61]. Choanoflagellates (*Monosiga brevicollis* and *Salpingoeca rosetta*) were selected as the outgroup for Timetree analysis when the RelTime method used evolutionary rates from the ingroup to calculate divergence times. The resulting phylogenetic tree was rendered using FigTree, providing a clear and visually interpretable representation of evolutionary relationships. A silicatein phylogenetic tree was constructed with four silicatein CDS (THG008206, THG008208, THG008209, and THG015260) from the *T. hoshinota* genome and the most closely related sequences, retrieved from the top 10 NCBI Blastp search hits. Phylogenetic analysis was done using the above procedure.

### Comparative analysis

To identify and compare the genomic content of specific domains or families related to development, cell adhesion molecules, and innate immunity, we used GO terms that were identified by InterProScan (-f tsv --goterms -dp) and compared the metabolism potential using KEGG orthology from six sponge genomes: *Amphimedon queenslandica* (GCA_000090795.2), *Sycon ciliatum* (GCA_964019385.1), *Corticium candelabrum* (GCA_963422355.1), *Dysidea avara* (GCA_963678975.2), *Halichondria panicea* (GCA_963675165.1), and *Oscarella lobularis* (GCA_947507565.1), and other lineage of animals: *Acropora millepora* (GCF_013753865.1), *Strongylocentrotus purpuratus* (GCF_000002235.5), *Drosophila melanogaster* (GCF_000001215.4), and *Homo sapiens* (GCF_000001405.40). These annotations were then compared to the genomic content of *T. hoshinota*, generating a list of target domains specific to each species for comparative analysis. In addition, a predominant cyanobacterial species genome, *Candidatus* Paraprochloron terpiosi (GCA_018141785.1) was retrieved to analysis metabolic interactions with *T. hoshinota* via KEGG GhostKOALA [62] annotation.

### Transcriptome analysis of heat and acid stress responses

*Terpios hoshinota* fragments (2–5 cm each) were collected from sponges covering *Montipora digitata* corals at Sesoko Beach, in October 2016. Five fragments were placed on each of 12 ceramic tiles, totaling 60 fragments, and acclimated in a flow-through Seawater tank at Sesoko Station for over 24 h before experiments.

#### Heat stress experiment

A heat-stress experiment was conducted in thermostated flow-through seawater tanks set to either control (27 °C) or heat stress (31 °C) conditions. Fragments were transferred from the acclimation tank to the experimental tanks, with three tiles per tank. Samples were collected on days 1, 4, and 8 after treatment. Prior to sampling, fragments were rinsed with sterilized water, cut into smaller pieces, and placed in 50-mL Falcon tubes containing 10 mL of TRIzol reagent (Thermo Fisher Scientific, USA). Samples were allowed to react with TRIzol™ reagent (Thermo Fisher Scientific, USA) at room temperature for 10–15 min before storage at − 80 °C.

#### Acid stress experiment

A acid stress experiment used flow-through seawater tanks adjusted to different pCO₂ levels: control (400 ppm) and acid stress (700 ppm) [63]. Fragments were transferred from the acclimation tank to the experimental tanks, with three tiles per tank. Samples were collected on days 1, 4, and 8 after treatment. As in the heat stress experiment, fragments were rinsed, cut into smaller pieces, and preserved in TRIzol reagent at − 80 °C after a 10–15-min reaction at room temperature.

#### RNA extraction and sequencing

Frozen sponge samples were thawed on ice and transferred to mortars containing 1.5 mL of TRIzol™ reagent. Samples were ground with liquid nitrogen until a fine powder was formed. The resulting sponge powder was transferred to 1.5-mL microcentrifuge tubes, and TRIzol™ was added as needed to achieve a final volume of 1.5 mL. Total RNA was extracted from sponge tissues using TRIzol™ reagent following the manufacturer’s protocol. RNA quality was then assessed with agarose gel electrophoresis.

RNA sequencing was conducted at the Genomics Core Facility of Academia Sinica. mRNA was isolated using oligo-dT magnetic beads, chemically fragmented, and reverse-transcribed into cDNA. Sequencing adapters were ligated, and cDNA fragments were amplified into clusters via PCR on a sequencing flow cell. Paired-end sequencing was performed on an Illumina NextSeq 2000, generating 101 bp x2 reads.

#### Differential expression gene analysis

RNA sequencing reads were aligned to the *T. hoshinota* genome assembled in-house using Bowtie2 [64]. Gene read counts were quantified with HTSeq-count [65], and the read count for each gene was used as a measure of its relative expression. To account for differences in sequencing depth and RNA composition among samples, read counts were normalized using the *median of ratios* method implemented in DESeq2 [66]. This normalization was applied to all samples from both the heat and acid stress experiments. Normalized read counts were subsequently considered as gene expression levels for downstream analyses. Genes differentially expressed under heat and acid stress conditions were identified using repeated-measurement ANOVA implemented in R with the aov function. Statistical significance was initially set at *p* < 0.05, and *p*-values were adjusted using the False Discovery Rate (FDR) correction with a significance threshold set at 0.1. However, no genes passed this FDR threshold; therefore, downstream enrichment analysis was performed based on the original (uncorrected) *p*-values. Gene Ontology (GO) enrichment analysis was conducted using the topGO [67] package in R, with separate analyses for upregulated (expression increased in the treated group) and downregulated (expression unchanged or decreased in the treated group) genes in Biological Process (BP) GO ontologies. Enrichment significance was assessed using the Fisher’s exact test implemented in topGO. All scripts used for differential expression and GO enrichment analyses are publicly available on GitHub (https://github.com/poyuliu/T_hoshinota_genome*).*

## Results

### Genome assembly and annotation of *Terpios hoshinota*

#### Genome size estimation and assembly

The genome size of *T. hoshinota* was estimated at 186.48 ± 4.30 Mbp using flow cytometry with rice (*Oryza sativa*) as a reference standard, based on triplicate measurements (Supplementary Figure S1). Genome assembly began with raw Nanopore reads, producing an initial draft of 3,882 contigs with total length 175,805,957 bp, and an N50 of 100,395 bp. Following four rounds of iterative polishing with Pilon and eukaryotic contig binning using EukRep to exclude prokaryotic contamination, the final assembly comprised 3,571 contigs spanning 169,428,171 bp with 131.475X average coverage, a contig N50 of 98,842 bp, and 40.85% GC content. BUSCO analysis confirmed high assembly quality with 97.4% genome completeness (Table 2), establishing a robust genomic resource for downstream analyses.

**Table 2:** Assembly statistics from the *T. hoshinota* genome

Assembly ver.	Contigs no.	≥ 10 kb contigs no.	Total length (bp)	≥ 10 kb length (bp)	Largest contig (bp)	Total length (bp)	GC (%)	N50 (bp)	BUSCO completeness
Assembly v.0^#^	3,882	2,866	175,805,957	170,607,576	3,835,627	175,805,957	41.11	100,395	-
Polish v.1^*^	3,882	2,866	175,656,470	170,463,308	3,839,872	175,656,470	41.11	100,307	-
Polish v.2^*^	3,882	2,863	175,598,682	170,380,274	3,838,089	175,598,682	41.11	100,339	-
Polish v.3^*^	3,882	2,861	175,574,271	170,339,385	3,838,093	175,574,271	41.11	100,288	-
Polish v.4^*^	3,882	2,861	175,572,499	170,337,498	3,838,091	175,572,499	41.11	100,339	-
EukRep^$^	3,571	2,811	169,428,171	164,894,299	1,653,179	169,428,171	40.85	98,842	97.4%

^#^draft assembly from raw ONT sequences by MetaFlye

^*^iterative polish for draft assembly by Pilon

^$^ eukaryotic contigs identification by EukRep

#### Annotation statistics

In total, 40,945 genes were predicted (Table 3). These predictions were further analyzed for functional annotation. Among the annotated genes, 24,761 were assigned to protein families using Pfam, while 27,905 genes were annotated against the NCBI NR database. Notably, 4,895 genes were categorized as hypothetical or uncharacterized proteins, and 13,040 were identified as unannotated open-reading frames (ORFs). These represent potential novel *T. hoshinota* genes.

**Table 3:** Annotation statistics from the *T. hoshinota* genome

Gene prediction model	CDS region (exon)	mRNA/gene no.	intron
GeneMark-ES	242,420	35,829	206,591
BRAKER3	269,517	40,945	229,903
Annotation models	Annotated genes	Hypothetical gene/Uncharacterized protein	Unannotated
KEGG/DIAMOND blastp			
KEGG Orthology (KO)	12,392	-	14,796
Pfam	24,761	-	2,427
Prot	12,382	NA/8	14,798
NCBI NR/DIAMOND blastp	27,905	4,895/5,121	13,040
Protein domain	Annotated domains	Uncharacterized domains	Unannotated
InterProScan Signature^#^	275,256	108/660	28,068
InterPro annotation	254,174	8/68	49,842
GO terms^*^	219,579 (5,047 terms)	-	183,964
Total annotated gene no.	28,610		

^#^Domain search databases Pfam, PANTHER, TIGRFAM, Gene3D, SMART, PRINTS, ProSiteProfiles, ProSitePatterns, HAMAP, PIRSF, SUPERFAMILY

^*^including multiple hits

Protein domain analysis revealed the functional diversity of the *T. hoshinota* genome. Using InterPro domain Searches, 275,256 unique protein signatures were identified. A total of 5,047 unique Gene Ontology (GO) terms related to molecular functions, biological processes and cellular components. Among these, *catalytic activity* (1,105 counts), *metabolic process* and *primary metabolic process* (1,233 and 1,011 counts), and *cellular anatomical entity* (727 terms) were the most prevalent (Table S1).

### Phylogenomic insights and evolutionary divergence

#### Phylogenetic position of *T. hoshinota*

**Fig. 3:**
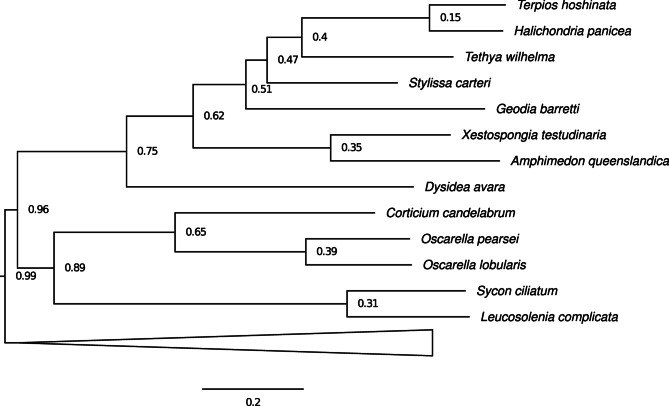
Species tree among poriferans inferred with OrthoFinder using 108 orthogroups. This phylogeny was reconstructed based on concatenated alignments of conserved orthologs, representing major lineages of Porifera. The species tree showed that *Terpios hoshinota* forms a sister group with *Halichondria panicea* among siliceous demosponges, with sequential divergence from other species in the Demospongiae and distinct outgroups, including *Dysidea avara*, *Corticium candelabrum*, *Oscarella* spp., *Sycon ciliatum*, and *Leucosolenia complicata*, highlighting both evolutionary and morphological divergence of the Porifera. Node support values were derived from OrthoFinder’s STAG algorithm

Phylogenomic analysis of sponge genomes revealed that *T. hoshinota* shares the closest evolutionary relationship with *Halichondria panicea* among siliceous sponges in the class Demospongiae, forming a sister group in the phylogenetic tree (Fig. 3). In the Demospongiae, *T. hoshinota* shows sequential divergence from *Tethya wilhelma*, *Stylissa carteri*, and *Geodia barretti*, while *Xestospongia testudinaria* and *Amphimedon queenslandica* form a separate sister clade. *Dysidea avara*, a keratose sponge lacking siliceous spicules, served as an outgroup to the entire Demospongiae clade. In addition to their phylogenetic divergence, these species exhibit notable morphological diversity. *T. hoshinota* displays a thin, encrusting form that spreads over coral surfaces, contrasting with the massive or barrel-shaped bodies of *X. testudinaria* and *G. barretti*, which possess prominent chimney-like oscula. Such morphological variation reflects distinct ecological strategies and may correspond to their evolutionary divergence within the Demospongiae. *Corticium candelabrum*, *Oscarella* spp., *Sycon ciliatum*, and *Leucosolenia complicata*, which do not belong to the Demospongiae clade do not share morphological features (Classes Homoscleromorpha and Calcarea). Next, we compared *T. hoshinota* with six reference sponge genomes in terms of development and metabolic potential, which reflect their ecological adaptation associated with evolutionary divergence.

#### Evolutionary timescale of *T. hoshinota*

**Fig. 4:**
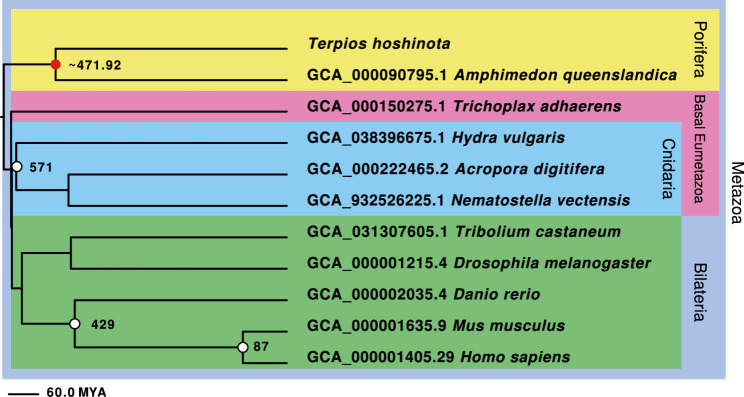
Phylogenomic relationships and divergence time of *T. hoshinota*based on single-copy orthologous genes. The evolutionary relationship of *T. hoshinota* in a phylogenomic framework was investigated using single-copy orthologous genes identified by OrthoFinder. Genomes from *T. hoshinota* and 12 other species, spanning Metazoa and Choanoflagellates (outgroup), were analyzed, with 20 shared single-copy genes concatenated for phylogenetic analysis. Maximum Likelihood phylogenetic analysis with the JTT model and a Timetree analysis using the RelTime method in MEGA 11 were performed, incorporating divergence time calibrations: *Homo sapiens*–*Mus musculus* (87 MYA, CI: 81.3–91.0 MYA), *Homo sapiens*–*Danio rerio* (429 MYA, CI: 423.3–440.0 MYA), and *Hydra vulgaris*–*Acropora digitifera* (571 MYA, CI: 543.0–610.0 MYA). Divergence of *T. hoshinota* and *Amphimedon queenslandica* was estimated at approximately 472 million years ago, during the Ordovician period

Phylogenomic analysis provided a detailed perspective on the evolutionary history of *T. hoshinota*. Utilizing 20 shared, single-copy, orthologous *T. hoshinota* genes, a sponge—*Amphimedon queenslandica*, 9 eumetazoan species, and two choanoflagellates as outgroups, this sponge was estimated to have diverged from *A. queenslandica* approximately 472 million years ago, during the Ordovician period (Fig. 4; the phylogenetic tree reconstructed from 196 orthogroups supported the inferred tree topology, see Supplementary Figure S3). Unlike the chimney-like structure of *A. queenslandica*, *T. hoshinota* exhibits a flat morphology. This unique structural adaptation may contribute to its invasive potential, enabling it to efficiently colonize coral reefs.

#### Phylogenetic analysis of spicules formation genes

**Fig. 5:**
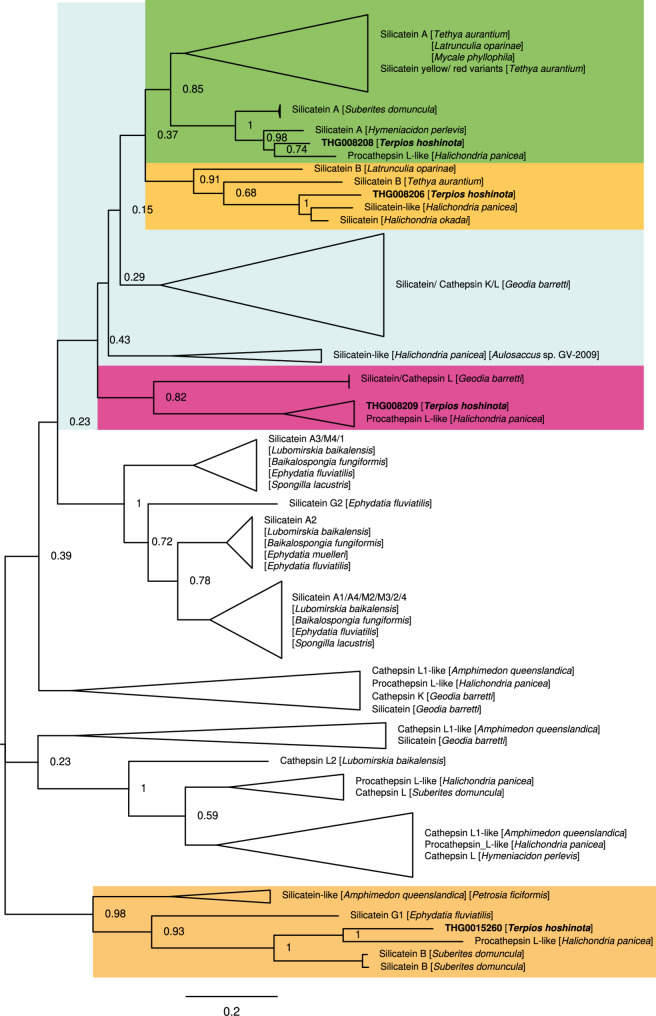
Phylogenetic relationships of silicatein genes in *T. hoshinota* and related sponges. A silicatein phylogenetic tree was constructed using four silicatein coding sequences (CDS) from the *T. hoshinota* genome (*THG008206*, *THG008208*, *THG008209*, and *THG015260*). Sequences from closely related species were retrieved from the top 10 hits in NCBI BLASTp searches. The tree highlights evolutionary relationships and diversification of silicatein genes, demonstrating similarities to homologs in other sponges

Silicatein genes, essential for biomineralization in sponges, were investigated to identify adaptations potentially linked to *T. hoshinota*’s distinctive flat morphology. Phylogenetic analysis, incorporating four silicatein coding sequences from *T. hoshinota* (THG008206, THG008208, THG008209, and THG015260) with homologous sequences from Demosponges, revealed both conserved features and unique diversification patterns (Fig. 5). The phylogenetic tree highlighted notable similarities between *T. hoshinota* silicateins and their homologs, including silicateins alpha, beta, and yellow variants (see Table S2), from sponges such as *Tethya aurantium* and *Hymeniacidon perlevis*. This demonstrates strong conservation of functional domains critical for spicule formation across Demosponges. However, one distant homolog (THG015260), annotated as silicatein beta from *Suberites domuncula*, displayed a distinctive evolutionary position on the phylogenetic tree. This divergence may indicate specialized adaptations associated with *T. hoshinota*’s ecological niche and morphology.

### Comparative analysis of functional domains adaptations associated with body plan

To identify unique adaptations in *T. hoshinota*’s body plan, we compared key differences in protein domain distributions linked to developmental processes, cell adhesion, and innate immunity, among seven sponge species representing major phylogenetic lineages. The comparative analysis is presented in Table 4 and S3, which present an overview of gene family variability obtained by comparing sequences identified in Interpro, and in Table S4, which was annotated using the KEGG database to categorize subclasses.

**Table 4:** Distributions of various protein domains and families associated with selected aspects of eukaryotic cell physiology in *T. hoshinota* and 6 sponge genomes

Process/Domain	Description	InterPro ID	Th	Hp	Aq	Da	Cc	Ol	Sc
Development									
Wnt	Wnt	IPR005817	8	3	5	11	12	19	33
HD	Homeodomain	IPR001356	58	38	41	38	61	66	109
Cell Adhesion Molecules									
Ig_I-set	Immunoglobulin I-set	IPR013098	156	197	109	381	257	316	210
Integrin_alpha	Integrin alpha chain	IPR000413	5	7	10	6	8	11	14
Integrin_bsu	Integrin beta subunit	IPR015812	18	9	14	11	10	3	17
Cadherin-like_dom	Cadherin-like	IPR002126	49	48	29	54	85	135	72
Cadherin	Cadherin	IPR039808	22	16	14	15	26	34	24
Selectin_CTLD	Selectin, C-type lectin-like domain	IPR033991	0	0	0	0	0	0	0
Selectin_superfamily	Selectin superfamily	IPR002396	0	0	0	4	0	0	4
Innate immunity									
TIR_dom	Toll/interleukin-1 receptor homology (TIR) domain	IPR000157	56	36	5	31	51	43	19
NLRP_innate_immun_reg	NLRP family, innate immunity and inflammation regulators	IPR050637	143	151	10	130	0	0	14
SRCR	SRCR domain	IPR001190	389	408	375	174	73	243	68

*Th* *Terpios_hoshinota*, *Hp* *Halichondria panicea*, *Aq* *Amphimedon queenslandica*, *Da* *Dysidea_avara*, *Cc* *Corticium candelabrum*, *Ol* *Oscarella_lobularis*, *Sc* *Sycon_ciliatum*

*T. hoshinota* and its closest phylogenetic relative, *H. panicea*, in the Demospongiae, with other sponges including *A. queenslandica* and *D. avara*, possess only NK-like genes (ANTP-like homeobox gene, NKL) and lack Hox-like genes (HOXL), reflecting the early evolution of metazoan genomes. However, *T. hoshinota* exhibits distinctive developmental gene features within this phylogenetic context, including three copies of Wnt6 genes (compared to zero in its sister species, *H. panicea* and the demosponge, *A. queenslandica*, but similar to *D. avara* with 2 copies). Notably, *T. hoshinota* shares Wnt11 genes with its phylogenetic sister species, *H. panicea* and *A. queenslandica*, whereas the calcareous sponge, *S. ciliatum*, shows the highest Wnt diversity (33 total copies), illustrating both conserved and lineage-specific evolution of these developmental regulators across sponge phylogeny.

*T. hoshinota* exhibits a prostrate, encrusting morphology, facilitating its extensive substrate coverage and aggressive overgrowth. This morphological adaptation is supported by abundant immunoglobulin-like domains (156 copies), which are intermediate among demosponges (*H. panicea*: 197, *A. queenslandica*: 109, *D. avara*: 381) but lower than the homoscleromorph *O. lobularis* (316 copies), and numerous cadherin-related domains (49 cadherin-like domains and 22 cadherin domains), enabling robust substrate attachment. The phylogenetically distinct calcareous sponge, *S. ciliatum*, and homoscleromorphs (*C. candelabrum*, *O. lobularis*) show markedly different integrin and cadherin distributions, reflecting their evolutionary divergence from the Demospongiae.

Furthermore, the aggressive coral-killing behavior of *T. hoshinota* is supported by enhanced cellular movement capabilities. Gene Ontology analysis reveals that *T. hoshinota* possesses the highest number of cell migration-associated genes (155 copies) among demosponges, closely matched by its phylogenetic sister, *H. panicea* (151 copies), and substantially higher than *A. queenslandica* (83 copies) (Table S5). *T. hoshinota* harbors moderate levels of positive regulators of cell migration genes and is similar to *A. queenslandica* (5 copies). The relatively low neuron migration gene content (3 copies) was comparable to those of other sponges in outgroup clades.

Lastly, encrusting sponges, such as *T. hoshinota* and *H. panicea* possesses flat morphologies and filter-feeding lifestyles, which increase their risk of exposure to environmental pathogens. We found that innate immunity-related domains show phylogenetically structured patterns: TIR domains in *T. hoshinota* (56 copies) and its sister species *H. panicea* (36 copies) are dramatically enriched compared to those in *A. queenslandica* (5 copies), whereas *D. avara* shows intermediate levels (31 copies). NLRP domains display similar phylogenetic clustering, with *T. hoshinota* (143 copies) and *H. panicea* (151 copies) showing comparable high abundance versus *A. queenslandica* (10 copies). Conversely, SRCR domain numbers show less phylogenetic structure, with *T. hoshinota* (389 copies), *H. panicea* (408 copies), and *A. queenslandica* (375 copies) displaying similar levels despite their evolutionary relationships, while the homoscleromorphs *C. candelabrum* (73 copies) and *O. lobularis* (243 copies) show reduced abundance, indicating both phylogenetic constraints and convergent adaptive responses in sponge immune systems. In addition to other lineage species in Table S3, encrusting sponges harbor more NLRP family domains than other invertebrates, such as cnidarians, echinoderms, and arthropods.

### Genomic complementarity from predominantly symbiotic cyanobacteria

In addition to innate immunity that may confer high tolerance to pathogens in sponges, *T. hoshinota* acquired a metabolically complementary symbiotic relationship with its predominant cyanobacterium, *Candidatus* Paraprochloron terpiosi LD05, particularly in biosynthesis of essential amino acids and vitamin B12 (Table S6). Comparative genomic analysis reveals significant differences in amino acid biosynthetic capabilities among sponge species: *T. hoshinota* and phylogenetic sister species *H. panicea* and *A. queenslandica* all lack biosynthetic pathways for arginine, asparagine, histidine, and lysine, whereas sponges *C. candelabrum*, *O. lobularis*, and *S. ciliatum* can synthesize these amino acids (except for lysine). On the other hand, like other animals, these sponge species completely lack both anaerobic and aerobic synthetic pathways for vitamin B12 biosynthesis. This indicates that sponges must rely on microorganisms to obtain this essential coenzyme. A gene content analysis of *Ca.* P. terpiosi and *T. hoshinota* suggest that a symbiotic, metabolic dependency exists between the sponge and its dominant cyanobacterial symbiont.

### Gene expression alteration of *T. hoshinota* under heat and acid stresses

**Fig. 6:**
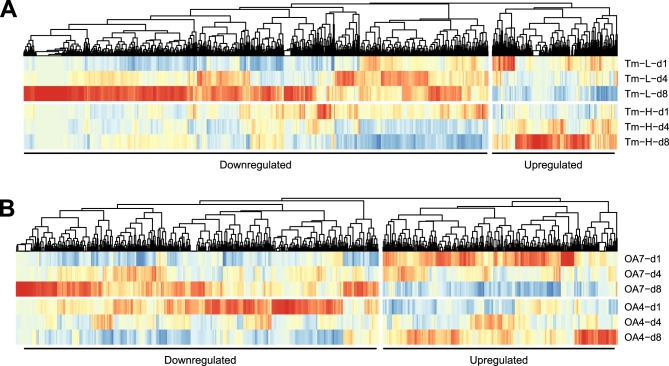
Gene expression changes in *T. hoshinota* under heat and acidification stress. RNA-seq analysis of *T. hoshinota* under heat and acidification stress reveals differentially expressed genes (DEGs) associated with stress-response mechanisms. DEGs were identified using repeated-measures ANOVA. **A** Gene expression under heat stress (31°C) was compared to control (27°C), with samples collected on days 1, 4, and 8. A total of 1,376 genes were upregulated (grouped into 53 significant Biological Process [BP] GO terms) and 371 genes were downregulated (grouped into 18 significant BP GO terms). Significant up- and downregulated BP GO terms are listed in Table S7. **B** Gene expression under acidification stress (700 ppm pCO₂) was compared to control (400 ppm pCO₂), with samples collected on days 1, 4, and 8. A total of 440 genes were upregulated and 285 genes were downregulated, each grouped into 13 significant BP GO terms. Significant up- and downregulated BP GO terms are listed in Table S7

We investigated plasticity of *T. hoshinota*’s gene expression machinery to cope with environmental stressors, especially climate change, providing insights into its resilience and invasive potential in fluctuating coral reef ecosystems. Gene expression profiles were analyzed under heat (31 °C) and acid stress (700 ppm pCO₂). RNA-seq analysis revealed observable trends in gene expression among key biological processes associated with stress responses. Differentially expressed genes (DEGs) were identified using repeated measurement ANOVA with a raw p-value < 0.05, although none passed the FDR < 0.1 cutoff (Fig. 6 and Table S7). Under heat stress, 1,376 genes were upregulated (grouped into 53 significant Biological Process [BP] GO terms, FDR < 0.05) and 371 genes were downregulated (grouped into 18 significant BP GO terms, FDR < 0.05). The most significantly upregulated processes included octopamine biosynthetic process, dopamine catabolic process, norepinephrine biosynthetic process, and gamma-aminobutyric acid signaling pathway, highlighting neurotransmitter metabolism and signaling responses to elevated temperatures. Downregulated processes were dominated by DNA duplex unwinding, protein K6-linked ubiquitination, and regulation of proteasomal protein catabolism, suggesting reduced DNA replication and protein degradation. Under acid stress, 440 genes were upregulated (grouped into 13 significant BP GO terms, FDR < 0.05) and 285 genes were downregulated (grouped into 13 significant BP GO terms, FDR < 0.05). Upregulated processes included tRNA C3-cytosine methylation, protein monoubiquitination, and sodium ion transport, reflecting cellular maintenance and ion homeostasis responses. Downregulated processes included mitophagy, tumor necrosis factor-mediated signaling pathway, and protein K63-linked ubiquitination, indicating suppressed autophagy and inflammatory responses in low-pH environments.

## Discussion

This study provides the first high-quality assembly and comprehensive annotation of the genome of *Terpios hoshinota*, a black coral-killing sponge that is threatening reef ecosystems. This genome is estimated at 186.48 Mbp and 40,945 genes were identified. A phylogenomic analysis estimated that *T. hoshinota* diverged from other sponge lineages during the Ordovician period (~ 472 million years ago), providing it with an extensive evolutionary history to develop unique structural and genetic traits. These adaptations likely underpin its invasive behavior and flat morphology. For instance, the *T. hoshinota* genome contains enriched domains for substrate adhesion, body plan development, innate immunity, and specialized silicatein genes. These genetic features may contribute to its environmental resilience and pathogen defense, enabling it to grow aggressively in reef ecosystems. Our work highlights *T. hoshinota*’s success as a coral reef invader. On the other hand, stress experiments under heat and acidification conditions demonstrated *T. hoshinota*’s plasticity, with upregulated pathways under heat stress enriched for neurotransmitter metabolism and signaling, including octopamine and norepinephrine biosynthesis, dopamine catabolism, and GABAergic signaling, while acid stress-induced pathways related to tRNA methylation, protein monoubiquitination, and sodium ion transport, reflect cellular maintenance and ion homeostasis responses. These insights into its resilience in fluctuating ecosystems support our genome annotation.

Despite its simple, sheet-like morphology, which suggest an ancient evolutionary origin, *T. hoshinota* belongs to the family Suberitidae, which is thought to have evolved relatively later than the earliest demosponges (class Demospongiae; 580 MYA) [68]. Suberitids exhibit both encrusting and massive growth forms, manifesting wide adaptation. However, due to the absence of hard skeletal structures, molecular fossil records such as 24-isopropylcholestane can better trace the origin of demosponges as far back as 635 MYA [69]. As a result, their origin and evolutionary timeline must be inferred from molecular markers in closely related extant species. Our phylogenomic analysis, which compared *Amphimedon queenslandica* (class Demospongiae, family Niphatidae) to *T. hoshinota*, estimated a divergence time of ~ 472 MY, corresponding to the Ordovician Period. This finding aligns with diversification of siliceous sponges during the Middle Ordovician [38, 70]. These phylogenomic insights pave the way for future studies to explore developmental processes, spicule formation, and innate immunity mechanisms in *T. hoshinota*, contributing to a deeper understanding of its evolutionary and ecological significance.

Spicules provide *T. hoshinota* with structural support, potentially serving as a defense mechanism. Their formation after larval settlement is influenced by environmental factors such as temperature and light [71]. Spicules contain silicatein, an enzyme that catalyzes polymerization of silicon [35, 72–75]. Spicule growth occurs through apposition of lamellar silica layers, with silicatein localized both on spicule surfaces and in their axial filaments [76]. In this study, we identified four types of silicatein genes in the *T. hoshinota* genome. Among these, three showed high homology with silicateins of closely related demosponges, such as *Tethya aurantium* and *Hymeniacidon perlevis*, suggesting conservation in morphology and function. For instance, in closely related sponges like *H. perlevis*, morphological variations are influenced by environmental changes: branched structures grow in sheltered areas, whereas convoluted or flat morphologies are observed in areas with wave action [77]. However, the fourth silicatein gene (THG015260) exhibited lower similarity to the other three. Its closest sequence match was found in *Suberites domuncula*, suggesting that *T. hoshinota*’s attachment behavior may share similarities with this species, which colonizes gastropod and crustacean shells [78].

We compared *Terpios hoshinota* with reference genomes of other sponges and metazoans, revealing consistency between morphological taxonomy and phylogenomic classification, which is also reflected in differences in protein domain numbers related to development, cell adhesion, and innate immunity (Table 4 and Table S3). Comparative analysis of functional domains revealed critical insights into the adaptive body plan of *T. hoshinota*, highlighting its flat morphology. Despite sharing similarities with other sponges, *T. hoshinota* possesses unique features, such as an additional Wnt6 gene, which contrasts with the broader Wnt diversification observed in vertebrates and highlights the evolutionary trajectory of Wnt signaling in sponges. Notably, *T. hoshinota* and *Homo sapiens* share enrichment of Wnt11 genes not generally seen in invertebrates, further illustrating the conserved yet specialized roles of these genes. Animal cell adhesion is intimately linked to Wnt signaling [79], and genes associated with cell adhesion molecules, such as immunoglobulin-like domains and cadherins, are abundant in *T. hoshinota*, providing evidence of functional diversification [80–82]. These molecules likely enhance *T. hoshinota’s* capacity to sense and adapt to environmental changes, facilitating its invasive success in reef ecosystems [21, 31]. Furthermore, by exploring the potential mobile ability of sponges through gene ontologies related to cellular and individual movement, which infer its invasive behavior, we found that *T. hoshinota* (Family Suberitidae) and *H. panicea* (Family Halichondriidae) possess highly similar motility-related genes (GO:0016477, cell migration with 155 and 151 copies, respectively) and individual morphology (flat, irregularly changing forms), despite their distinct geographic distributions [83].

On the other hand, development-related gene families, including homeobox (HOX) genes, exhibited notable duplication events in *T. hoshinota*, which are hypothesized to underpin its morphological specialization. However, the total number of homeodomain-containing genes in sponges, including *T. hoshinota*, is significantly lower than in other metazoans, consistent with findings from prior studies [39]. This disparity is consistent with observed expansion of homeodomain gene families through whole-genome duplications (WGDs) in higher vertebrates [84, 85], emphasizing the evolutionary significance of sponges as a basal lineage for understanding body plan evolution. The absence of Hox genes and the presence of NK-related genes in *T. hoshinota* suggest an essential function for NK genes in early metazoan evolution, supporting the hypothesis that ProtoHox genes originated from an NK-like ancestor [39, 86]. Additionally, innate immunity-related domains, including Toll/interleukin-1 receptor homology (TIR) and NACHT domains, are highly enriched in *T. hoshinota*. This notable enrichment indicates robust immune defense, enabling the sponge to effectively respond to microbial and environmental challenges, a characteristic commonly observed in other marine invertebrates [42, 87–89].High levels of scavenger receptor cysteine-rich (SRCR) domains, second only to those in the sea urchin, *Strongylocentrotus purpuratus* [90], underscore the sponge’s remarkable ability to sense and regulate interactions with its environment, suggesting that *T. hoshinota* possesses an innate immune system with a level of complexity that is comparable to that of other well-studied marine invertebrates.

Furthermore, comparisons with other sponges having more distant morphological and phylogenetic relationships, including *Amphimedon queenslandica* (*Aq*), *Dysidea_avara*, *Corticium candelabrum*, *Oscarella_lobularis*, and *Sycon_ciliatum*, showed distinct patterns in the number of cell and individual movement-related gene ontologies (These species belong to Homoscleromorpha and Calcarea, except for Aq). These genomes exhibited relatively high copy numbers for the following GO terms: GO:0048870—cell motility, GO:0001764—neuron migration, GO:0043542—endothelial cell migration, and GO:0030335—positive regulation of cell migration.

The enrichment of GO:0001764—neuron migration may reflect the presence of homologous genes related to neural synapses in sponge genomes. Although these genes do not form complete neuronal structures, they may have already formed ancient submodules with vesicle transport, calcium regulation, and synaptic scaffold functions. Studies indicated that basal animals such as *A. queenslandica* retain 125 homologous genes related to human synapses and exhibit modular co-expression characteristics in specific cell types and developmental stages [91]. These findings may support the basis of neural system evolution.

Additionally, previous studies on *D. avara* and *O. lobularis* regarding epithelial morphogenesis and regenerative capacity show that these primitive multicellular animals rely on existing epithelial cells for tissue remodeling and reconstruction, demonstrating extremely high cellular plasticity and transdifferentiation potential [92]. Particularly in *O. lobularis*, the outer epithelium can be reconstructed from multiple source cells without involving mesenchymal cell participation or blastema formation, indicating that regeneration is achieved primarily through epithelial sheet remodeling. These observations may correspond to the enriched GO term “GO:0043542—endothelial cell migration” found in this study, suggesting that cellular dynamic mechanisms similar to those in higher animals may already exist even in basal animal phyla.

Notably, both *T. hoshinota* and *H. panicea* form host-specific symbiotic relationships with specific symbiotic bacteria. Our analysis, along with previous work [19], shows that *T. hoshinota* may be nutritionally dependent on essential amino acids and vitamin B12 synthesized by its predominant symbiont, *Candidatus* Paraprochloron terpiosi LD05. However, genomic analysis of *H. panicea*’s dominant symbiont *Candidatus* Halichondribacter symbioticus by Knobloch et al. (2018) [93] revealed that genome reduction reflects metabolic dependence on the sponge (opposite to the case of *T. hoshinota*) and weakened DNA repair capacity to adapt to symbiosis.

Previous studies [18, 20] have indicated *T. hoshinota* may kill corals by blocking coral access to light and by using toxins. It has been hypothesized that the sponge physically overgrows and covers corals, reducing insolation for photosynthetic coral endosymbionts. However, this hypothesis still requires more direct physiological evidence. Interactions between *T. hoshinota* and corals (20 species) were examined by electron microscopy and showed no consistent morphological patterns among different corals covered by the same sponge [94], suggesting that such interactions depend upon the viability of both organisms. This observation also suggests that chemical effects are weak, but that physical blocking is more effective for killing corals. Accordingly, we examined mobility-related genes in *T. hoshinota* and other sponges. Indeed, we detected many more mobility-related genes (GO:0016477) in *T. hoshinota* and other encrusting sponges, and far fewer in immobile sponges. Although we do not fully understand how specific molecular mechanisms enable movement, these sponges clearly out-compete other organisms, such as corals. In addition, *T. hoshinota* does produce at least two toxins, nakiterpiosin and nakiterpiosinone [95]. Therefore, we also looked at potential pathways for alkaloid compounds. However, no relevant metabolic pathway was detected in *T. hoshinota*’s genome. Alkaloid toxins are commonly found in terrestrial plants, but are not detected in marine organisms, according to Teruya et al. [95]. Therefore, we speculate that these toxins may be produced by symbiotic cyanobacteria, not the sponge [19]. Symbiotic cyanobacteria would benefit the sponge in space competition with corals by releasing toxin. However, identifying genes or enzymes for toxin synthesis requires further investigation.

Furthermore, climate change-induced stress affects multiple physiological mechanisms in marine invertebrates, particularly those organisms inhabiting coral reef ecosystems [96, 97]. To assess the invasive potential and resilience of *T. hoshinota*, we simulated thermal (31 °C) and acidification (700 ppm pCO₂) stress. Under heat stress, we observed transcriptional upregulation of genes involved in neurotransmitter metabolism and signaling, such as octopamine, along with other biogenic amines such as dopamine, GABA, and norepinephrine. These amines mediate invertebrate responses to thermal stress by regulating metabolic rate, redox balance, and behavioral adaptation. Accumulating evidence across multiple taxa, including marine invertebrates, insects, and early-diverging metazoans, supports involvement of these neurotransmitters in stress resilience mechanisms and energy homeostasis during heat exposure [98–104].Concurrently, downregulation of genes related to DNA duplex unwinding and proteasomal degradation indicate reduced replication and protein turnover, possibly reflecting energy conservation strategies [105, 106] These shifts in transcriptional and epigenetic regulation may represent adaptive plasticity, allowing sponges to fine-tune gene expression for short-term thermal tolerance [107–109]. In contrast, under acidification, upregulated genes were enriched in processes such as tRNA cytosine methylation, monoubiquitination, and sodium ion transport, implying cellular efforts to maintain translational fidelity and ion homeostasis [110]. Downregulated genes were associated with mitophagy and TNF-mediated signaling, reflecting suppressed autophagic and immune pathways in low-pH environments. Interestingly, increased expression of DNA repair genes and initial upregulation of energy metabolism pathways suggest cellular damage responses and elevated metabolic demands during early acid stress [111–113]. Together, these stress-specific transcriptional responses highlight *T. hoshinota*’s capacity for physiological adjustment, which may confer competitive advantages in degrading reef environments and underscore its ecological threat to already stressed corals [110, 114–116].

In this study, we presented a high-quality genome assembly and comprehensive functional annotation of the encrusting sponge, *T. hoshinota*. Functional analysis and poriferan comparative genome analysis demonstrate that some special features of developmental regulation, cell adhesion, and innate immunity are involved. We identified key genomic features of cell migration underlying its invasive potential. Transcriptomic responses to heat and acidification stresses reveal its robust physiological plasticity in coping with environmental changes. In addition, *T. hoshinota*’s growth requires metabolic complementarity from its cyanobacterial symbionts. Our findings establish a molecular framework explaining the ecological invasion of this coral-killing sponge, facilitating future investigations of its impact on coral reefs.

## Supplementary Information

Supplementary Material 1: Supplementary Figure S1. Genome size of Terpios hoshinota, estimated by flow cytometry. Using rice (Oryza sativa) as a reference standard, the genome size of T. hoshinota was analyzed with a 488- nm laser under conditions of cell counts exceeding 100,000. An analysis of nucleic acid content, based on three independent measurements, determined the genome size to be 186.48 ± 4.30 Mbp. Region R2 was gated for analysis, with R3 representing the cumulative fluorescence signal of T. hoshinota.Supplementary Figure S2. PCR analysis of cyanobacterial contamination in the nucleus-like particle (NLP) working solution. Gel electrophoresis results indicate low levels of cyanobacterial contamination in the NLP solution, as detected using the cyanobacteria-specific 16S rRNA gene primer pair, CYA106F and CYA781R. M: DNA marker (100 bp ladder); P: Positive control (Escherichia coli DNA); NLP: Nucleus-like particle working solution; N: Negative control.Supplementary Figure S3. Species tree of various metazoans, inferred by OrthoFinder using 196 orthogroups. This phylogeny was reconstructed based on concatenated alignments of conserved orthologs, representing major metazoan lineages. Node support values were derived using OrthoFinder’s default STAG algorithm. This tree provides an evolutionary framework for comparative genomic analyses among metazoan taxa. Table S1 Top 10 GO slims of molecular function (MF), biological process (BP), and cellular component (CC) of Terpios hoshinota annotated genes. Table S2. Silicatein protein subtypes identified in T. hoshinota. Table S3. Distribution of various protein domains and families associated with selected aspects of eukaryotic cell physiology in T. hoshinota and representative animal genomes. Table S4. Distribution of various subclasses of protein domains associated with selected aspects of eukaryotic cell physiology in T. hoshinota and representative animal genomes, based on KEGG Orthology. Table S5. Comparison of motility-related genes (GO terms) among poriferan reference genomes. Table S6. Biosynthesis potential of essential nutrients (amino acids and vitamin B12) compared among Porifera reference and Ca. P. terpiosi LD50 (T. hoshinota’s symbiotic bacteria) genomes. Table S7. Significant Biological Process [BP] GO terms of differentially expressed genes in T. hoshinota under heat and acidification stress.

## Data Availability

Data from this study are publicly available. Sequencing data can be accessed at NCBI under BioProject accession number: PRJNA1231274. The complete genome sequence of T. hoshinota has been deposited in NCBI GenBank under accession number GCA_050230095.1. The code used for data analysis in this study is available at: https://github.com/poyuliu/T_hoshinota_genome.

## Abbreviations

TIR: Toll/interleukin-1 receptor
NLR: NOD-like receptors
SRCR: Scavenger receptor cysteine-rich
ML: Maximum likelihood
MYA: Million years ago
CDS: Coding squence
ONT: Oxford nanopore technology
GO: Gene ontology
NKL: NK-like
HOXL: Hox-like
DEGs: Differentially expressed genes
