# Supplementary material for: Genomic analysis reveals broad adaptability of coral-killing sponge (Terpios hoshinota) under environmental stress
Source: BMC Genomics. 2025 Sep 26;26:830. doi: 10.1186/s12864-025-11962-7 (PMC12465263; doi:10.1186/s12864-025-11962-7)
Supplement: Supplementary file 1 — Supplementary Material 1: Supplementary Figure S1. Genome size of Terpios hoshinota, estimated by flow cytometry. Using rice (Oryza sativa) as a reference standard, the genome size of T. hoshinota was analyzed with a 488- nm laser under conditions of cell counts exceeding 100,000. An analysis of nucleic acid content, based on three independent measurements, determined the genome size to be 186.48 ± 4.30 Mbp. Region R2 was gated for analysis, with R3 representing the cumulative fluorescence signal of T. hoshinota.Supplementary Figure S2. PCR analysis of cyanobacterial contamination in the nucleus-like particle (NLP) working solution. Gel electrophoresis results indicate low levels of cyanobacterial contamination in the NLP solution, as detected using the cyanobacteria-specific 16S rRNA gene primer pair, CYA106F and CYA781R. M: DNA marker (100 bp ladder); P: Positive control (Escherichia coli DNA); NLP: Nucleus-like particle working solution; N: Negative control.Supplementary Figure S3. Species tree of various metazoans, inferred by OrthoFinder using 196 orthogroups. This phylogeny was reconstructed based on concatenated alignments of conserved orthologs, representing major metazoan lineages. Node support values were derived using OrthoFinder’s default STAG algorithm. This tree provides an evolutionary framework for comparative genomic analyses among metazoan taxa. Table S1 Top 10 GO slims of molecular function (MF), biological process (BP), and cellular component (CC) of Terpios hoshinota annotated genes. Table S2. Silicatein protein subtypes identified in T. hoshinota. Table S3. Distribution of various protein domains and families associated with selected aspects of eukaryotic cell physiology in T. hoshinota and representative animal genomes. Table S4. Distribution of various subclasses of protein domains associated with selected aspects of eukaryotic cell physiology in T. hoshinota and representative animal genomes, based on KEGG Orthology. Table S5. Comparison of [file 12864_2025_11962_MOESM1_ESM.pdf]

Supplementary information

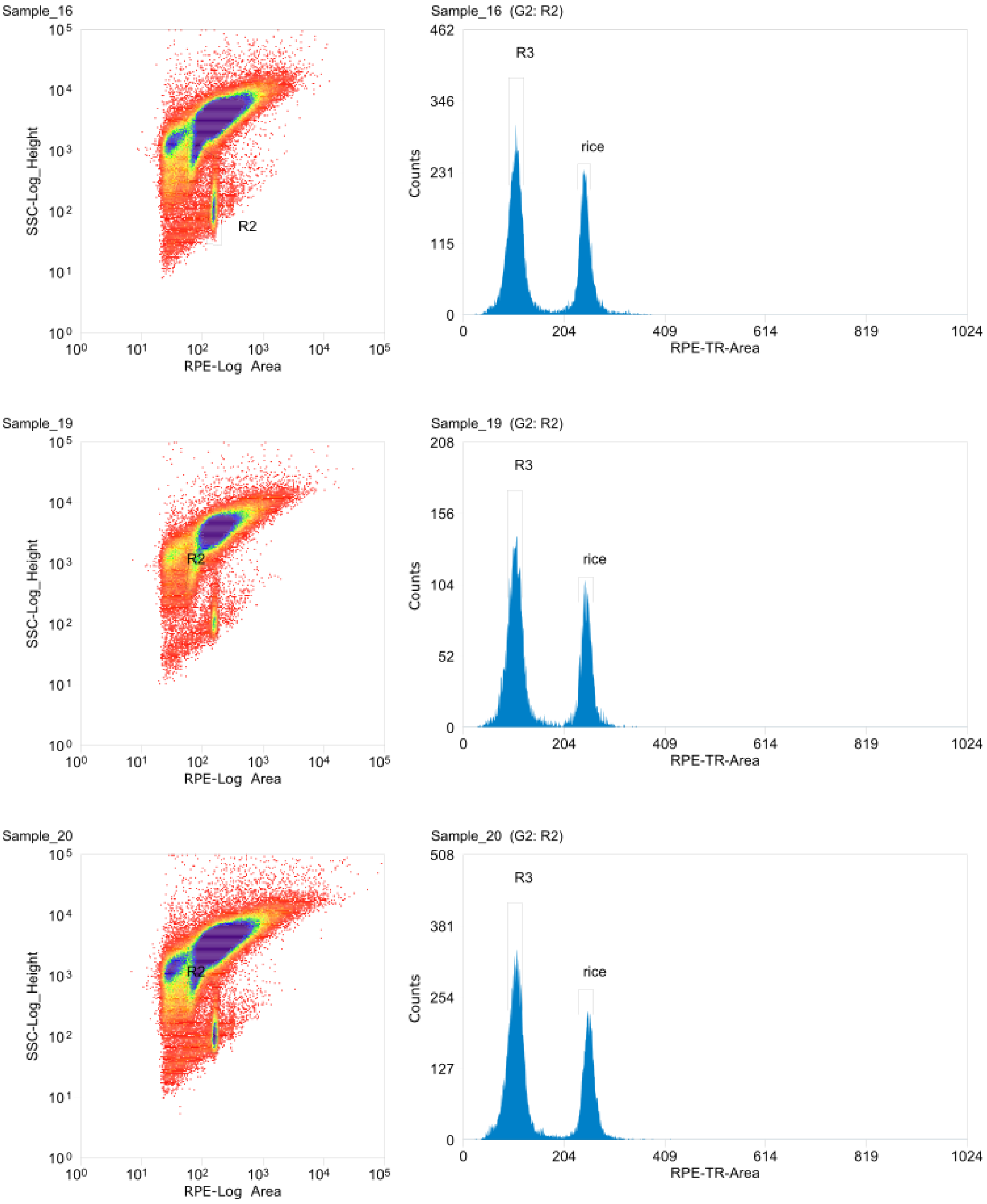

Supplementary Figure S1. Genome size of *Terpios hoshinota*, estimated by flow cytometry.

Using rice (*Oryza sativa*) as a reference standard, the genome size of *T. hoshinota* was analyzed with a 488-nm laser under conditions of cell counts exceeding 100,000. An analysis of nucleic acid content, based on three independent measurements, determined the genome size to be 186.48 ± 4.30 Mbp. Region R2 was gated for analysis, with R3 representing the cumulative fluorescence signal of *T. hoshinota*.

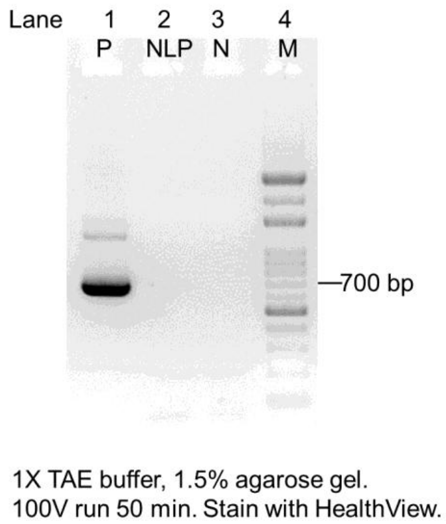

Supplementary Figure S2. PCR analysis of cyanobacterial contamination in the nucleus-like particle (NLP) working solution. Gel electrophoresis results indicate low levels of cyanobacterial contamination in the NLP solution, as detected using the cyanobacteria-specific 16S rRNA gene primer pair, CYA106F and CYA781R. M: DNA marker (100 bp ladder); P: Positive control (Escherichia coli DNA); NLP: Nucleus-like particle working solution; N: Negative control.

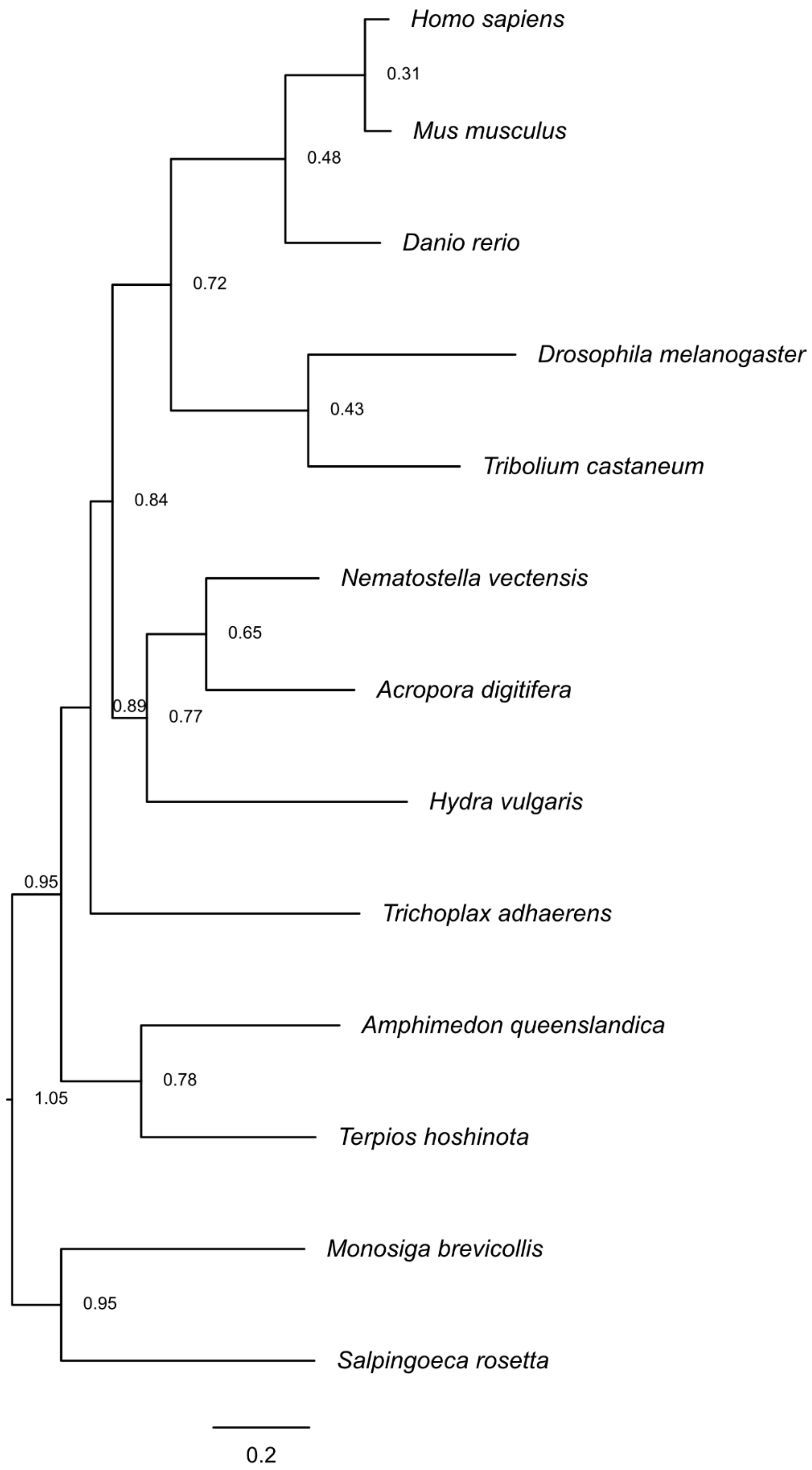

Supplementary Figure S3. Species tree of various metazoans, inferred by OrthoFinder using 196 orthogroups. This phylogeny was reconstructed based on concatenated alignments of conserved orthologs, representing major metazoan lineages. Node support values were derived using OrthoFinder's default STAG algorithm. This tree provides an evolutionary framework for comparative genomic analyses among metazoan taxa.

Table S1 Top 10 GO slims of *molecular function* (MF), *biological process* (BP), and *cellular component* (CC) of *Terpios hoshinota* annotated genes

**GO ontology**	**GO Term**	**Count**
MF	GO:0003824 catalytic activity	1105
MF	GO:0016740 transferase activity	413
MF	GO:0005488 binding	397
MF	GO:0016787 hydrolase activity	295
MF	GO:0016491 oxidoreductase activity	197
MF	GO:0005215 transporter activity	165
MF	GO:0005515 protein binding	161
MF	GO:0003676 nucleic acid binding	95
MF	GO:0016829 lyase activity	67
MF	GO:0016874 ligase activity	66
BP	GO:0008152 metabolic process	1233
BP	GO:0044238 primary metabolic process	1011
BP	GO:0043170 macromolecule metabolic process	691
BP	GO:0065007 biological regulation	663
BP	GO:0050789 regulation of biological process	581
BP	GO:0006139 nucleobase-containing compound metabolic process	495
BP	GO:0016043 cellular component organization	429
BP	GO:0051179 localization	379
BP	GO:0050896 response to stimulus	312
BP	GO:0051234 establishment of localization	310
CC	GO:0110165 cellular anatomical entity	727
CC	GO:0032991 protein-containing complex	527
CC	GO:0043226 organelle	512
CC	GO:0043229 intracellular organelle	483
CC	GO:0043231 intracellular membrane-bounded organelle	371
CC	GO:0005737 cytoplasm	274
CC	GO:0005634 nucleus	199
CC	GO:0016020 membrane	184
CC	GO:0043232 intracellular non-membrane-bounded organelle	168
CC	GO:0005856 cytoskeleton	67

Table S2. Silicatein protein subtypes identified in *T. hoshinota*.

Gene ID	Classification	KEGG Orthology	Prot	Pfam	Signature description	E-value	Subject description
THG008206	silicatein	K01365	CTSL; cathepsin L [EC:3.4.22.15]	Peptidase_C1 Inhibitor_I29 Peptidase_C1_2	Cysteine proteinases	5.63E-101	AAF21819.1 silicatein beta [Tethya aurantium]
THG008208	silicatein	K01365	CTSL; cathepsin L [EC:3.4.22.15]	Peptidase_C1 Inhibitor_I29 Peptidase_C1_2	Cysteine protease	1.80E-111	ABC94586.1 silicatein alpha [Hymeniacidon perlevis]
THG008209	silicatein	K01365	CTSL; cathepsin L [EC:3.4.22.15]	Peptidase_C1 Inhibitor_I29 Peptidase_C1_2	Cathepsin S	7.30E-98	CBY80149.1 silicatein yellow variant [Tethya aurantium]
THG015260	silicatein	blank	NA_value	Peptidase_C1 Inhibitor_I29 Peptidase_C1_2	Cathepsin S	1.65E-113	CAD67990.1 silicatein beta [Suberites domuncula]

Table S3. Distribution of various protein domains and families associated with selected aspects of eukaryotic cell physiology in *T. hoshinota* and representative animal genomes.

Process/Domain	Description	InterPro ID	Th	Aq	Am	Sp	Dm	Hs
**Development**								
Wnt	Wnt	IPR005817	4	5	56	17	11	65
HD	Homeodomain	IPR001356	57	42	242	178	260	953
**Cell Adhesion Molecules**								
Ig_I-set	Immunoglobulin I-set	IPR013098	138	109	320	182	329	1467
Integrin_alpha	Integrin alpha chain	IPR000413	4	10	6	12	14	115
Integrin_bsu	Integrin beta subunit	IPR015812	14	14	2	6	6	76
Cadherin-like_dom	Cadherin-like	IPR002126	44	29	67	40	49	422
Cadherin	Cadherin	IPR039808	18	14	31	18	22	167
Selectin_CTLD	Selectin, C-type lectin-like domain	IPR033991	0	0	0	0	0	9
Selectin_superfamily	Selectin superfamily	IPR002396	0	0	0	3	0	9
**Innate immunity**								
TIR_dom	Toll/interleukin-1 receptor homology (TIR) domain	IPR000157	49	5	111	271	28	169
NLRP_innate_immun_reg	NLRP family, innate immunity and inflammation regulators	IPR050637	127	10	70	0	0	140
SRCR	SRCR domain	IPR001190	356	375	95	639	10	132

Th: *Terpios hoshinota*, Aq: *Amphimedon queenslandica*, Am: *Acropora millepora*, Sp: *Strongylocentrotus purpuratus*, Dm: *Drosophila melanogaster*, Hs: *Homo sapiens*

Table S4. Distribution of various subclasses of protein domains associated with selected aspects of eukaryotic cell physiology in *T. hoshinota* and representative animal genomes, based on KEGG Orthology.

Process	Description	Th	Hp	Aq	Da	Cc	Ol	Sc	Am	Sp	Dm	Hs
**Development**												
Wnt	WNT4	2	1	1	3	3	3	12	4	2	0	1
	WNT6	3	0	0	2	0	0	0	1	1	1	1
	WNT7	1	1	3	1	0	6	4	3	1	0	2
	WNT11	1	1	1	0	0	0	1	0	0	0	1
Homeodomain	ANTP: HOXL	0	0	0	0	0	0	1	14	16	17	52
	ANTP: NKL	6	5	5	11	7	7	9	32	24	18	48
	Paired-related	6	6	5	6	15	5	6	29	30	15	61
	LIM	4	5	4	6	12	9	9	7	8	8	15
	POU	3	4	9	3	2	2	12	5	6	4	19
	SINE	2	2	2	1	2	2	3	3	3	3	6
	ZF	0	0	0	0	0	0	0	0	2	2	12
	CUT	2	1	1	1	3	2	4	2	4	2	7
	PROS	0	0	0	0	0	0	0	0	2	1	2
	TALE	3	3	0	1	6	7	15	4	7	8	20
**Cell Adhesion Molecules**												
Immunoglobulin	Immunoglobulin superfamily	10	19	9	56	64	84	59	31	22	6	55
Integrins	Integrins	12	7	5	4	21	13	26	5	11	1	26
Cadherins	Cadherins	39	36	15	53	63	71	53	10	12	9	113
Selectins	Selectins	26	27	0	30	5	6	60	0	4	0	3

Sponge species—Th: Terpios_hoshinota, Hp: Halichondria_panicea, Aq: Amphimedon queenslandica, Da: Dysidea_avara, Cc: Corticium candelabrum, Ol: Oscarella_lobularis, Sc: Sycon_ciliatum. Other species—Am: *Acropora millepora*, Sp: *Strongylocentrotus purpuratus*, Dm: *Drosophila melanogaster*, Hs: *Homo sapiens*

Table S5. Comparison of motility-related genes (GO terms) among poriferan reference genomes.

GO ID	GO.term.name	Th	Hp	Aq	Dy	Cc	Ol	Sc
GO:0016477	cell migration	155	151	83	120	74	65	107
GO:0048870	cell motility	8	8	7	11	18	14	19
GO:0006935	chemotaxis	-	-	1	8	-	1	-
GO:0001764	neuron migration	3	5	6	28	16	19	11
GO:0043542	endothelial cell migration	1	1	1	3	6	7	2
GO:0030335	positive regulation of cell migration	5	11	5	6	8	9	1
GO:0030336	negative regulation of cell migration	3	3	7	5	3	5	5
GO:0040011	locomotion	2	2	1	1	1	1	1

Th: Terpios_hoshinota, Hp: Halichondria_panicea, Aq: Amphimedon queenslandica, Da: Dysidea_avara, Cc: Corticium candelabrum, Ol: Oscarella_lobularis, Sc: Sycon_ciliatum.

Table S6. Biosynthesis potential of essential nutrients (amino acids and vitamin B12) compared among Porifera reference and *Ca.* P. terpiosi LD50 (*T. hoshinota*'s symbiotic bacteria) genomes.

**Amino acid**	Th	Hp	Aq	Dy	Cc	Ol	Sc	LD05
Ala	+	+	+	+	+	+	+	-
Arg	-	-	-	-	+	+	+	+
Asn	-	-	-	+	+	+	+	+
Asp	+	+	+	+	+	+	+	+
Cys	+	+	+	+	+	+	+	+
Glu	+	+	+	+	+	+	+	+
Gln	+	+	+	+	+	+	+	+
Gly	+	+	+	+	+	+	+	+
His	-	-	-	-	+	+	+	+
Ile	+	+	+	+	+	+	+	+
Leu	+	+	+	+	+	+	+	+
Lys	-	-	-	-	-	-	-	+
Met	+	+	+	+	+	+	+	+
Phe	-	-	-	-	-	-	-	-
Pro	+	+	+	+	+	+	+	+
Ser	+	+	+	+	+	+	+	+
Thr	+	+	+	+	-	-	+	+
Trp	-	-	-	+	-	-	-	+
Tyr	+	+	+	+	+	+	+	-
Val	+	+	+	+	+	+	+	+

**Anaerobic Cobalamin biosynthesis**	Th	Hp	Aq	Dy	Cc	Ol	Sc	LD05
cysG	-	-	-	-	-	-	-	-
cobA	-	-	-	-	-	-	-	+
cobA-hemD	-	-	-	-	-	-	-	+
MET8	-	-	-	-	-	-	-	-
sirC	-	-	-	-	-	-	-	-
cbiK	-	-	-	-	-	-	-	-
cbiX	-	-	-	-	-	-	-	+
cfbA	-	-	-	-	-	-	-	-
cobI-cbiL	-	-	-	-	-	-	-	+
cobJ	-	-	-	-	-	-	-	+
cbiGH-cobJ	-	-	-	-	-	-	-	+
cbiH60	-	-	-	-	-	-	-	-
cobM	-	-	-	-	-	-	-	+
cbiG	-	-	-	-	-	-	-	-
cbiGH-cobJ	-	-	-	-	-	-	-	+
cbiD	-	-	-	-	-	-	-	+
cobK-cbiJ	-	-	-	-	-	-	-	+
cbiT	-	-	-	-	-	-	-	+
cbiE	-	-	-	-	-	-	-	-
cobL-cbiET	-	-	-	-	-	-	-	+
cobH-cbiC	-	-	-	-	-	-	-	+
cobB-cbiA	-	-	-	-	-	-	-	+

**Aerobic Cobalamin biosynthesis**	Th	Hp	Aq	Dy	Cc	Ol	Sc	LD05
cobA	-	-	-	-	-	-	-	+
cobA-hemD	-	-	-	-	-	-	-	+
cobI-cbiL	-	-	-	-	-	-	-	+
cobIJ	-	-	-	-	-	-	-	+
cobG	-	-	-	-	-	-	-	-
cobJ	-	-	-	-	-	-	-	+
cobIJ	-	-	-	-	-	-	-	+
cbiGH-cobJ	-	-	-	-	-	-	-	+
cobM	-	-	-	-	-	-	-	+
cobF	-	-	-	-	-	-	-	-
cobK-cbiJ	-	-	-	-	-	-	-	+
cobL-cbiET	-	-	-	-	-	-	-	+
cobH-cbiC	-	-	-	-	-	-	-	+
cobB-cbiA	-	-	-	-	-	-	-	+
cobN	-	-	-	-	-	-	-	+
cobS	-	-	-	-	-	-	-	-
cobT	-	-	-	-	-	-	-	-

Th: Terpios_hoshinota, Hp: Halichondria_panicea, Aq: Amphimedon queenslandica, Da: Dysidea_avara, Cc: Corticium candelabrum, Ol: Oscarella_lobularis, Sc: Sycon_ciliatum. LD05: Candidatus Paraprochloron terpiosi LD05.

Table S7. Significant Biological Process [BP] GO terms of differentially expressed genes in *T. hoshinota* under heat and acidification stress.

Upregulated GO under heat stress

GO.ID	Term	Annotated	Significant	Expected	weightFisher	Ontology
GO:0006589	octopamine biosynthetic process	21	6	0.94	0.00023	BP
GO:0042420	dopamine catabolic process	21	6	0.94	0.00023	BP
GO:0042421	norepinephrine biosynthetic process	21	6	0.94	0.00023	BP
GO:0007214	gamma-aminobutyric acid signaling pathwa...	113	14	5.04	0.00046	BP
GO:0007186	G protein-coupled receptor signaling pat...	277	26	12.36	0.0005	BP
GO:0030224	monocyte differentiation	2	2	0.09	0.00198	BP
GO:0006564	L-serine biosynthetic process	2	2	0.09	0.00198	BP
GO:2000369	regulation of clathrin-dependent endocyt...	3	2	0.13	0.00577	BP
GO:0045747	positive regulation of Notch signaling p...	3	2	0.13	0.00577	BP
GO:0051046	regulation of secretion	21	4	0.94	0.01111	BP
GO:0006470	protein dephosphorylation	133	12	5.93	0.01521	BP
GO:0071577	zinc ion transmembrane transport	13	3	0.58	0.01801	BP
GO:0006468	protein phosphorylation	222	15	9.9	0.01831	BP
GO:0046512	sphingosine biosynthetic process	6	2	0.27	0.02641	BP
GO:0006882	intracellular zinc ion homeostasis	7	2	0.31	0.03589	BP
GO:0034587	piRNA processing	7	2	0.31	0.03589	BP
GO:0006643	membrane lipid metabolic process	72	9	3.21	0.04383	BP
GO:0051028	mRNA transport	22	2	0.98	0.0446	BP
GO:0019243	methylglyoxal catabolic process to D-lac...	1	1	0.04	0.04461	BP
GO:0008298	intracellular mRNA localization	1	1	0.04	0.04461	BP
GO:0099044	vesicle tethering to endoplasmic reticul...	1	1	0.04	0.04461	BP
GO:0006616	SRP-dependent cotranslational protein ta...	1	1	0.04	0.04461	BP
GO:0071233	cellular response to leucine	1	1	0.04	0.04461	BP
GO:1990253	cellular response to leucine starvation	1	1	0.04	0.04461	BP
GO:0140300	serine import into mitochondrion	1	1	0.04	0.04461	BP
GO:0033499	galactose catabolic process via UDP-gala...	1	1	0.04	0.04461	BP
GO:0042989	sequestering of actin monomers	1	1	0.04	0.04461	BP
GO:0009098	leucine biosynthetic process	1	1	0.04	0.04461	BP
GO:0050728	negative regulation of inflammatory resp...	1	1	0.04	0.04461	BP
GO:1903232	melanosome assembly	1	1	0.04	0.04461	BP
GO:0010165	response to X-ray	1	1	0.04	0.04461	BP
GO:0140021	mitochondrial ADP transmembrane transpor...	1	1	0.04	0.04461	BP
GO:0010142	farnesyl diphosphate biosynthetic proces...	1	1	0.04	0.04461	BP
GO:0106300	protein-DNA covalent cross-linking repai...	1	1	0.04	0.04461	BP
GO:0035229	positive regulation of glutamate-cystein...	1	1	0.04	0.04461	BP
GO:0006011	UDP-glucose metabolic process	1	1	0.04	0.04461	BP
GO:0051694	pointed-end actin filament capping	1	1	0.04	0.04461	BP
GO:1901029	negative regulation of mitochondrial out...	1	1	0.04	0.04461	BP
GO:0015811	L-cystine transport	1	1	0.04	0.04461	BP
GO:0009104	lipopolysaccharide catabolic process	1	1	0.04	0.04461	BP
GO:1990810	microtubule anchoring at mitotic spindle...	1	1	0.04	0.04461	BP
GO:0070301	cellular response to hydrogen peroxide	1	1	0.04	0.04461	BP
GO:1901031	regulation of response to reactive oxyge...	1	1	0.04	0.04461	BP
GO:0016031	tRNA import into mitochondrion	1	1	0.04	0.04461	BP

GO:0033211	adiponectin-activated signaling pathway	1	1	0.04	0.04461	BP
GO:0090160	Golgi to lysosome transport	1	1	0.04	0.04461	BP
GO:0140206	dipeptide import across plasma membrane	1	1	0.04	0.04461	BP
GO:0043457	regulation of cellular respiration	1	1	0.04	0.04461	BP
GO:1990544	mitochondrial ATP transmembrane transpor...	1	1	0.04	0.04461	BP

Downregulated GO under heat stress

GO.ID	Term	Annotated	Significant	Expected	weightFisher	Ontology
GO:0006891	intra-Golgi vesicle-mediated transport	40	4	0.24	9.10E-05	BP
GO:0032508	DNA duplex unwinding	55	4	0.33	0.0042	BP
GO:0085020	protein K6-linked ubiquitination	1	1	0.01	0.0061	BP
GO:1902103	negative regulation of metaphase/anaphas...	2	1	0.01	0.0121	BP
GO:0061136	regulation of proteasomal protein catabo...	32	2	0.19	0.0177	BP
GO:0055070	copper ion homeostasis	3	1	0.02	0.0181	BP
GO:0018022	peptidyl-lysine methylation	3	1	0.02	0.0181	BP
GO:0010950	positive regulation of endopeptidase act...	3	1	0.02	0.0181	BP
GO:2000009	negative regulation of protein localizat...	3	1	0.02	0.0181	BP
GO:0006268	DNA unwinding involved in DNA replicatio...	38	2	0.23	0.022	BP
GO:0034085	establishment of sister chromatid cohesi...	4	1	0.02	0.0241	BP
GO:0015014	heparan sulfate proteoglycan biosyntheti...	5	1	0.03	0.03	BP
GO:0070475	rRNA base methylation	5	1	0.03	0.03	BP
GO:1902306	negative regulation of sodium ion transm...	5	1	0.03	0.03	BP
GO:0090263	positive regulation of canonical Wnt sig...	5	1	0.03	0.03	BP
GO:2000045	regulation of G1/S transition of mitotic...	5	1	0.03	0.03	BP
GO:0030050	vesicle transport along actin filament	6	1	0.04	0.0359	BP
GO:0018401	peptidyl-proline hydroxylation to 4-hydr...	6	1	0.04	0.0359	BP
GO:0031398	positive regulation of protein ubiquitin...	7	1	0.04	0.0418	BP

Upregulated GO under acidification stress

GO.ID	Term	Annotated	Significant	Expected	weightFisher	Ontology
GO:0106217	tRNA C3-cytosine methylation	1	1	0.01	0.0091	BP
GO:0070197	meiotic attachment of telomere to nuclea...	1	1	0.01	0.0091	BP
GO:0006513	protein monoubiquitination	18	2	0.16	0.0114	BP
GO:0006814	sodium ion transport	39	3	0.36	0.0123	BP
GO:0055070	copper ion homeostasis	3	1	0.03	0.0271	BP
GO:0090309	positive regulation of DNA methylation-d...	3	1	0.03	0.0271	BP
GO:2000009	negative regulation of protein localizat...	3	1	0.03	0.0271	BP
GO:0006470	protein dephosphorylation	133	4	1.21	0.0324	BP
GO:0006122	mitochondrial electron transport, ubiqui...	4	1	0.04	0.036	BP
GO:0051026	chiasma assembly	4	1	0.04	0.036	BP
GO:1904491	protein localization to ciliary transiti...	4	1	0.04	0.036	BP
GO:1902306	negative regulation of sodium ion transm...	5	1	0.05	0.0448	BP
GO:0048268	clathrin coat assembly	5	1	0.05	0.0448	BP

Downregulated GO under acidification stress

GO.ID	Term	Annotated	Significant	Expected	weightFisher	Ontology
GO:0000423	mitophagy	3	1	0.02	0.016	BP
GO:0006814	sodium ion transport	39	2	0.21	0.019	BP

GO:0033209	tumor necrosis factor-mediated signaling...	42	2	0.23	0.022	BP
GO:0070534	protein K63-linked ubiquitination	42	2	0.23	0.022	BP
GO:0006122	mitochondrial electron transport, ubiqui...	4	1	0.02	0.022	BP
GO:0016560	protein import into peroxisome matrix, d...	5	1	0.03	0.027	BP
GO:0036371	protein localization to T-tubule	5	1	0.03	0.027	BP
GO:0055117	regulation of cardiac muscle contraction	5	1	0.03	0.027	BP
GO:0048240	sperm capacitation	5	1	0.03	0.027	BP
GO:0030317	flagellated sperm motility	6	1	0.03	0.032	BP
GO:1903566	positive regulation of protein localizat...	6	1	0.03	0.032	BP
GO:0006470	protein dephosphorylation	133	3	0.72	0.035	BP
GO:0034427	nuclear-transcribed mRNA catabolic proce...	7	1	0.04	0.037	BP
